# Reviewed article: Teixeira MMP, Santos DF, Barros EA, Santos LH, Siqueira LS, Pascoal LM, Costa ACPJ, Neto MS. Factors associated with deaths due to visceral leishmaniasis among children at a hospital in the southwest of Maranhão state: a cross-sectional study, Imperatriz, 2010-2021. Epidemiol Serv Saude. 2025:34;e20240738

**DOI:** 10.1590/S2237-96222025v34e20240738.a

**Published:** 2025-09-29

**Authors:** Allan Batista Silva

**Affiliations:** 1 Universidade Federal da Paraíba, João Pessoa, PB, Brazil Universidade Federal da Paraíba João Pessoa PB Brazil

## Peer review A

## Questionnaire

Does the manuscript contain new and significant information to justify publication? Yes

Does the Abstract (Summary) clearly and accurately describe the content of the article? Yes

Is the problem significant and concisely stated? Yes

Are the methods described comprehensively? Yes

Are the interpretations and conclusions justified by the results? Yes

Is adequate reference made to other work in the field? Yes

## Manuscript Structure

Length of article is: Adequate

Number of tables is: Adequate

Number of figures is: Adequate

Please state any conflict(s) of interest that you have in relation to the review of this paper. None

Interest: Good

Quality: Average

Originality: Average

Overall: Average

Recommendation: Accept

## Comments

O artigo é interessante e apresenta uma temática de importância pública, apesar de ser uma doença negligenciada. Diante disso, sugiro a publicação para que trabalhos como esse sejam divulgados e para que as doenças negligenciadas, como a leishmaniose, seja cada dia mais investigada.

